# Prognostic factors associated with locally recurrent rectal cancer following primary surgery (Review)

**DOI:** 10.3892/ol.2013.1640

**Published:** 2013-10-23

**Authors:** YANTAO CAI, ZHENYANG LI, XIAODONG GU, YANTIAN FANG, JIANBIN XIANG, ZONGYOU CHEN

**Affiliations:** Department of General Surgery, Huashan Hospital, Fudan University, Shanghai 200040, P.R. China

**Keywords:** rectal carcinoma, local recurrence, risk factors, prognosis

## Abstract

Locally recurrent rectal cancer (LRRC) is defined as an intrapelvic recurrence following a primary rectal cancer resection, with or without distal metastasis. The treatment of LRRC remains a clinical challenge. LRRC has been regarded as an incurable disease state leading to a poor quality of life and a limited survival time. However, curative reoperations have proved beneficial for treating LRRC. A complete resection of recurrent tumors (R0 resection) allows the treatment to be curative rather than palliative, which is a milestone in medicine. In LRRC cases, the difficulty of achieving an R0 resection is associated with the post-operative prognosis and is affected by several clinical factors, including the staging of the local recurrence (LR), accompanying symptoms, patterns of tumors and combined therapy. The risk factors following primary surgery that lead to an increased rate of LR are summarized in this study, including the surgical, pathological and therapeutic factors.

## 1. Introduction

Locally recurrent rectal cancer (LRRC) is a complication that occurs following primary rectal cancer resection. The introduction of total mesenteric excision (TME) has standardized the surgical technique in treating primary rectal cancer and has greatly reduced the local recurrence (LR) rate from 30% (1), in the pre-TME era, to 10% as reported (2). Although progress has been made in decreasing the LR rate, it remains a problem that is associated with a poor quality of life and short survival time. In the early years, the LRRC disease state was considered to represent the terminal stage. LRRC was considered a contraindication for curative surgical intervention, with surgeries only performed as palliative methods to relieve tumor-related symptoms, including obstruction and hemorrhage. Limited improvements to this prognosis have been noted with palliative adjuvant chemoradiotherapy (CRT) only. As experience accumulated, radical surgical resection was introduced into clinical practice in a select group of LRRC patients. Data from numerous centers have been collected and analyzed, and a curative reoperation has been shown to be the only chance to achieve a curative effect. The surgical safety and post-operative quality of life were declared acceptable. Although a curative surgical approach may be provided in patients with good systemic and local tumor conditions, the majority of patients do not qualify and have already lost the chance of curative surgery when diagnosed with LRRC. Even in carefully selected patients who underwent salvage surgery, the results have shown that less than half finally achieved a radical resection (3). Studies have identified factors that may cause a high risk of LR. Certain factors may be prevented during the treatment of the primary tumor. Furthermore, an early diagnosis of LRRC in patients with these factors is the most effective and economic method to ensure a curative reoperation. The present study reviewed the literature to analyze the significant risk factors that lead to LR following primary rectal cancer resection. The relevant factors affecting the prognosis following a resection of LRRC are also discussed in this study.

## 2. Recurrent risk factors following primary tumor resection

### Primary surgical factors

#### Location of primary tumor

A low tumor location was defined as rectal cancer below the level of peritoneal reflection, which was considered to be an independent factor affecting the risk of LR in certain studies (4), but this remains controversial (1). As it is not covered by the peritoneum, rectal cancer of the lower segment has a more extensive local infiltrative behavior than a tumor in the upper segment. Improved intraoperative exposure and easier surgical manipulation also contribute to a better prognosis with a lower LR rate (2,5).

#### Surgical type

Patients treated with a primary abdominoperineal resection (APR) were noted to have a higher rate of LR than those who underwent a low anterior resection (LAR) (6). Discussions led to the belief that selection bias may explain this difference. In primary rectal cancer cases, bulky, extensive and poorly-differentiated tumors in the lower part of the rectum usually require an APR procedure to ensure oncological safety. Although a poorer prognosis is noted, APR is the most suitable procedure in certain extensive low rectal cancer cases and in the majority of the ultralow cases (7). Indications for a sphincter-preserving procedure in low rectal cancer patients should be carefully handled, since an inappropriate ultralow anterior resection in advanced cases may significantly increase the risk of LR (8). The newly developed intersphincteric resection (ISR) expanded the sphincter-preserving options for low and ultralow rectal cancer patients. With careful selection of the early stage patients and with control of the surgical indications, no significant differences are observed compared with traditional APR in terms of surgical outcome (morbidity, mortality, LR rate and median survival time), according to a recent systematic review (9) With a limited number of ISR cases noted, more evidence is required to support this conclusion.

The choice of surgical type is based on a pre-operative assessment of several factors, including tumor staging, location and intrapelvic tumor invasion. The surgical type affecting the rate of LR should be the pathological pattern of the primary tumor.

#### R-stage

Following the resection of a primary rectal cancer, R-stage is defined by the post-operative residual tumor situation with regard to the specimen. The R-stage is divided into three levels: R0 stands for no residual tumor confirmed microscopically; R1 indicates microscopical tumor residue; and R2 indicates macroscopical tumor residue. An R0 resection is considered curative surgery, while R1 and R2 resections are palliative procedures, earning a median life expectancy of ~30 months and a much higher risk of LR and distal metastasis (10). Although adjuvant CRT may have a compensatory effect, there is a marked difference between the prognosis of R0 and R1/R2 tumors. This R-stage system is also suitable for evaluating the outcome of LRRC surgery.

#### Perforation

An intraoperative perforation of a tumor is generally regarded as a severe problem to the prognosis. In perforation cases, an increased risk of local tumor cell spreading results in degradation of the tumor staging and has been shown to be an independent factor for local and distant recurrence (11). In the majority of cases, perforations were accompanied by an inadequate excision of tissue surrounding the tumor or an incomplete tumor resection. The 5-year LR rate was noted to be 29% with perforation and 10% without perforation, as demonstrated by a Norwegian study (12). Prevention methods include pre-operative CRT, appropriate types of surgical procedures and an increased intraoperative focus of surgeons.

### Pathological factors

#### Patterns of pathology

The pathological examination of surgical resection specimens is critical in assessing the risk of recurrence. The associated pathological factors include the tumor stage, histological differentiation, extramural penetration, tumor border configuration and tumor budding (13,14). Data from several large clinical trials indicated that the most significant pathological risk factors for LRRC were a positive circumferential resection margin (CRM), lymphovascular invasion, advanced T-stage, extramural venous invasion and poor tumor differentiation (15,16,17). The importance of evaluating CRM is one of the most significant factors. Pathologists play a key role in assessing an accurate CRM determination. Ignorance of CRM involvement may lead to a negative pathological report, thus misdirecting the expectations of the prognosis. The specimens should be carefully dealt with and be sampled at the closest possible distance from the tumor and positive lymph node to the CRM. The majority of researchers tend to consider angioinvasion as an adverse risk factor for LR (18). However, this is inconsistent with the follow-up data recorded by Lee *et al*, although a significant difference was observed between distant metastasis and overall survival (19).

#### CRM

The surgical resection of rectal cancer has been standardized by the worldwide introduction of TME, which was first described in 1986 (20). With the mesorectum removed, the assessment of CRM becomes important and should be indicated in the pathological report. A positive CRM is defined as tumor extension and positive lymph nodes within 1 mm of the CRM, and this concept has been widely accepted. An LR rate of 37% was reported by Gosens *et al* in a case of CRM involvement, together with a rate of 8% in the CRM-free cases from a group of 201 patients (21), while the rate was 22 vs. 5% in a study from a Norwegian Center (n=686) (22,23). A CRM of ≤2 mm confers a poorer prognosis, as indicated by a study by Nagtegaal *et al*(24), and patients with this criteria are recommended neoadjuvant treatment based on the results by Bernstein *et al*(25). The CRM cut-off point leading to a high risk of LR was set at 1 mm in the majority of studies. In the study by Nagtegaal *et al*(24), patients with a positive CRM caused by tumor extension had a lower chance of LR than those with a positive CRM due to positive lymph nodes, but the P-value narrowly missed reaching statistical significance (22.1% vs. 12.4; P=0.06) (3).

Although critical in predicting the chance of recurrence, improvements in surgical technique and multidisciplinary therapy allow CRM to be more controllable. Pre-operative radiotherapy has also been introduced as neoadjuvant therapy for resectable rectal cancer and this results in a decreased LR rate, as shown by certain large multicenter studies (26,27). Neoadjuvant and adjuvant therapy may partly offset the poor prognosis in CRM-positive patients. Neoadjuvant therapy decreases the risk of a positive CRM prior to surgery, while adjuvant procedures contribute to the prevention of LR following a positive CRM being diagnosed. In a study by Kang *et al*, which consisted of 499 patients, no significant differences were observed for the LR rate between the CRM-positive and CRM-negative groups (13.0 and 13.5%, respectively; P=0.677) (28).

In CRM-negative cases, a complete resection of the mesorectum is also confirmed to be an indicator of the TME surgical quality. Several factors, including the surgeon’s technique and local tumor invasion, have an effect on the complete mesorectum resection. Leite *et al* reported 25% LR at 5 years, with incomplete mesorectum resections during the primary surgery. This value was 10% when the mesorectal excision was adequate (29). Application of the TME principle is the key factor in decreasing the LR rate.

#### Distal resection margin

Sphincter preservation and oncological safety are conflicting factors prior to choosing a type of surgery. Decisions are based on a balance, but assurance of radicality should always be the golden rule. The initial principle of TME requires the incision to be 5 cm distal to the inferior aspect of the tumor to avoid deposits of tumor cell being left. In the past few decades, the safe distal margin has been gradually shortened from 5 cm to 5 mm (30,31). Developments in adjuvant CRT and neoadjuvant CRT (nCRT) have provided chances to challenge the limit of the distal margin. A further understanding of regional lymphovascular anatomy and evidence based medicine aids in the renewal of the minimal safe distal margin (32). In a recent systematic review concerning the value of a 1-cm distal resection margin, 17 studies were included, with a distal margin of >1 cm in 948 patients compared with a margin of >1 cm in 4,626. The review concluded that in the selected group of patients, a negative distal margin of <1 cm did not significantly jeopardize oncological safety and was acceptable in distal rectal cancer cases (33). A marked regression from the fresh specimen to a formalin-fixed status was observed, and this made the pathological report of the distal resection margin conservative. To achieve a more accurate result and avoid biases of regression, surgeons should learn to assess the distal margin from fresh specimens on the operating table.

#### Carcinoembryonic antigen (CEA) level

CEA has been widely used as clinical tumor marker. By tracking patients who have undergone radical surgery, the continuous process of monitoring serum CEA levels is useful in forming an LR diagnosis. Young *et al* evaluated 236 patients who underwent radical surgery and identified an association between a high post-operative CEA level and a poor prognosis (high recurrence rate and poor survival) (34). Also, a decrease from relatively high pre-operative CEA levels to low post-operative levels usually indicates a good tumor radicality and predicts a good prognosis. In another large perspective study with 1,361 post-operative rectal cancer cases enrolled in Taiwan, the pre-operative high level CEA cases that retained high post-operative levels were reported to experience more frequent recurrence and metastasis (35). During follow-up visits subsequent to the primary rectal surgery, CEA monitoring is valuable to aid in predicting the prognosis and the recurrence risk.

### Therapeutic factors

#### Adjuvant CRT and nCRT

A Swedish systematic review analyzed 42 clinical trials and three meta-analyses containing a total of 26,351 patients (36). The review indicated that pre-operative radiotherapy may reduce the rate of LR by 50–70%, compared with a 30–40% reduction with post-operative radiation alone in primary local advanced rectal cancer cases. Another systematic review from the Rochester Center confirmed the beneficial effect of neoadjuvant therapy, the indication of which was set at stage II and III locally advanced rectal cancer by the TNM staging system (37). 5-Fluorouracil based chemotherapy in neoadjuvant and adjuvant settings was added to radiotherapy to overcome the dose limitations and enhance the effect of reducing the LR rate (38). Combined CRT also revealed an improved outcome in controlling LR and distal metastasis. By contrast, a poor reaction under exposure of nCRT was another risk factor for a poor prognosis (39).

## 3. Factors affecting prognosis when LRRC is diagnosed

### Pretheraputic factors of LRRC

#### Type of initial surgery

Patients who underwent an initial LAR were noted to have improved resectability and longer survival times compared with the initial APR when LRRC was diagnosed (40). The early diagnosis of LRRC in primary LAR cases is mostly facilitated by a digital rectal examination or fibrocolonoscopy during the follow-up period or by symptoms of bleeding or bowel habit changes. For primary APR patients, the earliest complaint to confirm a diagnosis of LRRC is usually pelvic pain, which may indicate severe intrapelvic invasion. Difficulty in forming an early diagnosis is a significant cause of the poorer prognosis in primary APR LRRC patients. Furthermore, the lack of a protective effect by the rectum and mesorectum enables a quick invasion from the LR site into neighboring organs or structures, which directly leads to a decrease in resectability (41). To achieve an R0 resection in these patients, an extended resection procedure is required and is accompanied by an increased rate of complication and short-term mortality.

#### Staging of LRRC

Wanebo *et al* suggested a classification scheme for LR based on the primary rectal cancer TNM staging system. Tr1 is classified as an LR invading the submucosa or limited muscularis at the primary resection site, while Tr2 is defined as a full thickness invasion of the muscularis propria. Tr3 is a full thickness penetration into the perirectal soft tissue. LR with an extensive invasion into an adjacent organ is classified as Tr4. Tr5 cases are an LR with extensive pelvic invasion. Intrapelvic recurrence following APR is directly sorted into Tr5 (42).

A study by the Mayo clinic first introduced the LRRC staging system based on the number of fixation sites to the pelvic wall (43). Fixation indicates advance recurrence, increases surgery difficulty and deteriorates the prognosis of LRRC compared with the mobile tumors in the study by Hahnloser *et al*(44).

Distant metastasis combined with LRRC is common according to reviews of the literature. The majority of studies regarded this situation as a contraindication for further curative surgery, and palliative therapy was usually provided. Whether LR was resectable or unresectable showed no difference in median survival time when distant metastasis was detected in LRRC patients (45).

#### Accompanied symptoms

Pelvic pain is the most common symptom that is associated with pelvic recurrence, which may radiate to the lower extremities. Bleeding and bowel habit changes are usually the only complaint prior to the detection of LRRC. The presence of symptoms is significantly associated with the resectability of LRRC. Symptomatic patients with LR usually have a more extensive tumor development followed by a lower chance of R0 resection than asymptomatic cases. This may explain the poorer prognosis following a reoperation (46). When the curative reoperation is accomplished, the response of symptomatic relief is regarded as a positive prognostic factor (35).

### Theraputic factors of LRRC

#### Surgical factors

Surgical treatment for LRRC is no longer considered a palliative procedure, but a curative intention due to the improvements in the techniques, experience and development of nCRT.

Caricato *et al* reviewed literature between 1982 and 2004 and indicated that an R0 resection is the most critical factor affecting the prognosis following a surgical intervention (47). This conclusion was confirmed by a subsequent study (48). Data collected from the Norwegian Colorectal Cancer Registry, which contained data on 577 LRRC patients treated between 1993 and 2001, also confirmed that an R0 resection is the most significant prognostic factor in treating recurrent rectal cancer. In this study, the 5-year overall survival rate was 55% following an R0 resection and 20% following an R1 resection (23). Although regarded as a failed radical resection resulting in a poorer prognosis compared with an R0 resection, microscopically-positive resection margin cases result in a longer survival time compared with palliative or untreated cases. Results from several studies concerning the distribution of R-stage and survival states following LRRC salvage surgery are presented in Table I.

#### Patterns of recurrence

The location of the recurrence site has proved to be a crucial factor affecting surgical radicality and resulting in various levels of surgical difficulty and tumor invasion. A study by Wiig *et al* indicated that 70–80% of LRRC following primary LAR was derived from perirectal tissue and appeared at the level near the anastomosis (55). The isolated recurrent site from the anastomosis was defined as the central type, also known as the anastomosis type. As summarized by Moore *et al*, patients with central site recurrence are identified to have the highest rate of radicality, while presacral LR has the poorest surgical outcome (56). Other sites of recurrence result in a similar rate of R0 resection of ~60%. When a complete resection was achieved, no statistical difference existed between the survival and recurrence rates among the anatomical types (57).

Anastomotic LRRC behaves as the most localized of all the LRRC types. Relatively improved intraoperative vision and less adjacent organ invasion may assist in achieving a radical resection. Recurrence involving the lateral pelvic side wall has the worst resectability potential and prognosis from clinical experience. At the cost of increased surgical trauma, morbidity and a decreased quality of life, the anterior type of LR with adjacent organ invasion (bladder, uterus, prostate) requires an extended combined organ resection to achieve radicality, while sacrectomy is widely applied in presacral LR. Essentially, it is the site-related surgical difficulty of oncological clearance that mainly causes the difference in radicality. The involvement of the iliac vessels and the pelvic side wall are considered unlikely to reach an R0 resection (58).

#### High sacrectomy

As concluded from the surgical experiences of several centers, presacral LRRC is usually associated with a large tumor volume and invasion into the sacrum. Increased surgical risks and complications have been reported, particularly during a curative abdominotranssacral resection. However, it may be the only type that has the potential to achieve a radical excision with invasion of the bony pelvic wall in contrast with the invasion of the anterior and lateral pelvic sidewalls, which are almost impossible to radically resect (44). Sacrectomy has proved its value in treating a posterior type of LR, with satisfied survival rates and affordable morbidity. An involvement level above the S2/S3 junction has for a long time been defined as a contraindication of this type of surgery in order to avoid injury to the spinal nerves and ensure the stability of the pelvis. However this concept may be changed in the near future (59). Experiences of performing high sacrectomy have been accumulated in several studies. According to a recent systemic review with 33 studies, the safety level was raised to the S1/S2 level in nine studies and to L5/S1 in one study (60). The post-operative R0 life expectancy was close to other surgical types for LRRC, but a poor quality of life limited the clinical application of this surgical type. The maximum load of the bony pelvis decreased to one-third of the normal structure for cases in which sacrum below the S1/S2 junction was resected (61). The level of the high sacrectomy and the associated information with regard to the long-term consequences of this procedure should be addressed pre-operatively and at follow-up visits (62). Scientists are making efforts in the biochemical field to develop new reconstruction techniques using myocutaneous flaps or artificial materials following a sacrectomy (63). A larger cohort of patients is required for further establishment of oncological safety.

#### Adjuvant and nCRT

LRRC treated with surgery alone has reported unsatisfying prognoses, with a median survival time of approximately seven-eight months and a 5-year overall survival of 0%. Multidisciplinary treatment is increasingly being used to improve the clinical outcome. Dassanayake *et al* used a multi-factorial linear logistic model for analyzing LRRC patients who did not undergo pre-operative CRT, and the result supported the significance of nCRT in rectal cancer (64). nCRT was indicated to have a suppressive effect on neural invasion in the study by Ceyhan *et al*(65). At present, the use of multidisciplinary treatment in LRRC patients is mostly extrapolated from past experiences of treating primary advanced rectal cancer cases. Further studies are required to confirm this aggressive method of treatment with curative intention.

As palliative therapy, a total dose of 45 Gy is usually administered to LR patients who are unsuitable for surgical intervention, with the aim of relieving their pain rather than radically treating the tumor. The median duration of symptom control has been recorded as approximately five months, with a survival duration of six months, as discussed in a Korean study (66).

#### Intraoperative radiation therapy (IORT)

IORT provides three times the biological effect of external beam radiotherapy, with a reduced dose of irradiation and with the normal structure shielded from the irradiation field. The toxicity complications may also be decreased. These advantages provide IORT with a more efficient therapeutic effect and less toxicity. The dose of IORT is adjusted as a 15–30 Gy boost to a specific area (67,68). Guo *et al* reported a three-year overall survival rate of 43% (69). Another study from the Mayo Center with 123 patients treated with surgery and IORT reported a three-year survival rate of 39% and a five-year survival rate of 20%, which was significantly higher than for those who were not administered IORT (70). However, data obtained from a study by Dresen *et al* did not record any difference in a comparison between patients with and without IORT (71). One negative point hindering the popularization of IORT is the costly equipment and complex manipulation. Further experience and clinical trials are required to understand whether IORT aids LRRC patients.

## 4. Summary and future

LRRC is no longer defined as a termination of treatment, but is now a potentially curable disease process. Multidisciplinary treatment, the radical resection of LR in conjunction with adjuvant, nCRT and other combined approaches are the current dominant treatment trends. With regard to a curative aim, an R0 resection is the root of success to be emphasized in this system in a carefully selected group of patients. Further investigation with regard to LR risk factors is critical in preventing recurrence when treating the primary rectal cancer. In high-risk patients, more frequent follow-up visits may contribute to an early diagnosis of LR and therefore expand the group of patients who are fit for curative surgery. Developments in CRT may also aid in increasing the chance of performing R0 resections in the near future.

## Figures and Tables

**Table I:** Distribution of R-stage following LRRC salvage surgery.

First author, year (ref.)	Patients, n	R0, n (%)	5-year OS, %	R1, n (%)	5-year OS, %	R2, n (%)	5-year OS, %
Pacelli *et al*, 2010 (49)	44^a^	28 (64)	72.4	16 (36)	37.5		
Jiang *et al*, 2011 (50)	187	87 (47)	42.6	60 (32)	17.2	40 (21)	0
Rahbari *et al*, 2011 (51)	92	54 (59)	70^b^	25 (27)	26^b^	13 (14)	11^b^
Hansen *et al*, 2009 (23)	577^c^	97 (52)	55	88 (48)	20		
Kusters *et al*, 2009 (57)	70^d^	92 (54)	40.5				
Heriot *et, al*, 2008 (52)	152	98 (61)	46	40 (26)	22	14 (9)	16
Asoglu *et al*, 2007 (48)	50	24 (48)	40^b^	12 (24)	18^b^	14 (28)	0^b^
Harji *et al*, 2013 (53)	30	10 (33)	77^b^	15 (50)	27^b^	5 (17)	0^b^
Wiig *et al*, 2011 (54)	124	52 (42)	53	51 (41)	19	21 (18)	0

LRRC, locally recurrent rectal cancer; OS, overall survival.

a Only R0 and R1 were taken into account in this study.

b Three-year survival rate.

c 185 patients from a total of 577 underwent a curative resection (R0/R1), the others were treated with pallitive radiotherapy with or without a surgical process.

d Only the surgical outcome of an R0 resection was shown in this study.
